# What is required to scale-up and sustain biofortification? Achievements, challenges and lessons from scaling-up Orange-Fleshed Sweetpotato in Sub-Sahara Africa

**DOI:** 10.1016/j.jafr.2021.100102

**Published:** 2021-06

**Authors:** Godfrey Mulongo, Hilda Munyua, Adiel Mbabu, Joyce Maru

**Affiliations:** a Value Plus Consultants, Box 28950, 00100, Nairobi, Kenya; b International Potato Center, Box 25171, 00603, Nairobi, Kenya; c Manconsult Limited, Kenya

**Keywords:** Capacity development, Orange-fleshed sweetpotato, Policy, Scaling-up

## Abstract

This review presents results of the ex-post survey on Reaching Agents of Change (RAC) project, highlighting experiences, lessons, challenges and recommendations for scaling up orange-fleshed sweetpotato (OFSP). The RAC project was a three-and halfyear initiative (2011 and 2015), implemented in three primary countries, namely Tanzania, Mozambique, Nigeria, and to a lesser extent Ghana and Burkina Faso. The project advocated for policy change and increased investments to scale-up the orangefleshed sweetpotato (OFSP) to combat vitamin A deficiency. RAC planned to generate new investments totaling US$ 18 million for OFSP activities in the three years of its life but exceeded this target by 20%. RAC further expected to benefit at least 600,000 households directly and is currently on track, having reached 309,974 direct beneficiaries (of whom 20.3% were women). The RAC experience demonstrated a potential scaling-up model for biofortified crops based on the hypothesis that scaling up can be achieved through supportive policies (and investments), strong institutional capacities and appropriate innovative technologies working through a partnership of governmental and non-governmental organizations and civil society.

## Highlights

• Article presents a potential scaling-up model for technologies in agricultural research and development (AR4D) projects.
• Organic institutional capacity building, strong institutions and inclusive policy formulation is efficacious in scaling-up.
• With limited resources, a cascaded training model can facilitate comprehensive institutional capacity building.
• Innovative technology supply models are necessary to cover diverse client conditions in smallholder agriculture.
• Public-private partnerships can catalyse effective value chain development.
• Integrating advocacy in public policy process yields more responsive policies for the smallholder agricultural producers.
• Scaling is thus a function of effective institutions, supportive policies and sustainable supply of technologies.

## Graphical abstract

**Figure:**
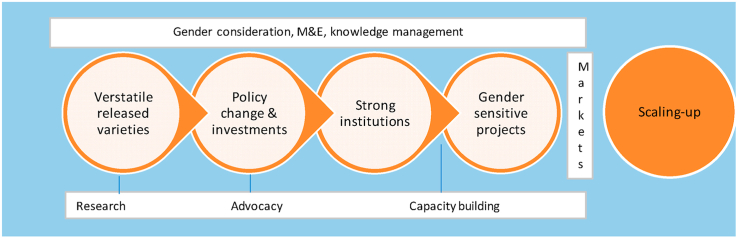

## 1 Background

Vitamin A deficiency (VAD) is of public health concern worldwide [1]. It is estimated that globally about 30% of children under 5 years of age are vitamin A deficient, and about 2% of all deaths are attributable to VAD in this age group [2]. In addition, an estimated 250,000 to 500,000 vitamin A-deficient children become blind every year, half of them dying within 12 months of losing their sight. VAD is caused by a habitual diet that provides too little bioavailable vitamin A to meet the physiologic needs [3]. Rapid growth and frequent infections, which cause ineffective utilization of the vitamin, are also critical factors [4]. Governments and their development partners have been relying on dietary diversification, food fortification and vitamin A supplementation to control the problem [5].

While vitamin A supplementation and industrial food fortification has helped millions [6], its delivery is expensive in isolated rural areas, which is where the poor live, and is difficult to sustain [5]. Many foods that are good sources of vitamin A such as fruits, vegetables, meat, milk and fortified foods such as margarine, sugar and oil are only available seasonally, unpalatable for young children or too expensive for the majority of rural people who are at most risk of VAD [7]. Orange-fleshed sweetpotato (OFSP) is an emerging cheap source of vitamin A particularly for remote rural areas where individuals have limited access to commercial markets and depend on crops produced at the household level [8]. OFSP has the advantage of fortifying itself by loading high levels of minerals and vitamins in its roots and leaves. A 100-g serving, or about half a cup, of the boiled roots can supply the daily vitamin A requirements of a young child (400 retinol activity equivalents (RAEs)) and thereby help to eliminate or greatly reduce VAD [8]. OFSP is rich in not only beta-carotene but also vitamins B and C and iron [9,10]. The OFSP roots are eaten raw, boiled or roasted as a substitute for bread during breakfast, while the leaves are an important relish taken with different staples [11].

### 1.1 OFSP penetration and trajectory in Sub-Sahara Africa (SSA)

The history of OFSP in SSA is well summarized in Low et al. [8]. Hotz et al. (2011, 2012), Brauw et al. (2015), Grüneberg et al. (2015) and Andrade et al. (2016) have also extensively published research highlighting preliminary and contemporary work conducted by the Consortium of International Agricultural Research Centers (CGIAR) and government research institutions with funding from bilateral and multilateral donors.

The introduction of OFSP in SSA dates to the early 1990s. In 1995, with funding from the International Center for Research on Women, the International Potato Center (CIP) and the Kenya Agriculture Research Institute (KARI) began OFSP research as part of a broader effort to develop and test women-focused approaches for addressing VAD. The willingness to fund research of that nature was very low because the international community's focus was on vitamin A capsule supplementation [10]. There was lack of evidence on food-based approaches as a remedy for VAD. Most of the sweetpotato efforts by CGIAR research institutions were limited to breeding OFSP varieties in Peru and sending them to SSA for evaluation. Several new OFSP varieties bred in Peru arrived in SSA in 2002 but performed poorly under the high virus pressure conditions. In settings with low virus pressure like Mozambique OFSP varieties were promising, and in April 1999 the first multisectoral stakeholder meeting was organized to promote OFSP utilization.

Only two SSA countries, i.e. Uganda and South Africa, were actually breeding sweetpotato in the early 1990s. The Ugandan national program started breeding OFSP in 1991. The McKnight Foundation provided consistent financial support to the Ugandan program from 1994 through 2014, enabling Uganda to lead in OFSP breeding in East and Central Africa [10]. Around this period, investment in OFSP remained low due to the lack of a strong evidence base supporting investment in OFSP [12]. In cognizance of this, OFSP efficacy studies were conducted in South Africa, Mozambique and Uganda between 2002 and 2005, and they showed significant improvements in vitamin A intake in children involved in the studies [13].

Together, the efficacy studies and the success of OFSP as a disaster-response crop for the devastating floods that occurred in Mozambique from February to March 2000, led to investments by the United States Agency for International Development (USAID) and the Government of Mozambique for disseminating OFSP as part of the development efforts in some provinces of Mozambique from 2002 through 2006 [10].

Other notable milestones for OFSP SSA include: a 4- year initiative funded by the Rockefeller Foundation in 2005 for breeding initiative in Mozambique, which ultimately led to the release of 15 drought-tolerant OFSP varieties by 2011 (Andrade et al., 2016b) and; a 5-year US$ 22.5 million project funded in 2009 by the Bill & Melinda Gates Foundation. This CIP led project, Sweetpotato Action for Security and Health in Africa (SASHA) supported establishment of advanced breeding in three sub-regions to address virus resistance, drought tolerance, tuber quality (aiming for a non-sweet sweetpotato), seed systems research, and research on delivery models.

### 1.2 OFSP status in Mozambique, Tanzania and Nigeria

At the inception of RAC, Mozambique, Tanzania and Nigeria were at different levels of development with respect to OFSP activities. For example, owing to the work started in the early 1990s, Mozambique released eight varieties of OFSP in 2001, and in 2005 it received from the Rockefeller Foundation support for an additional four years for breeding, which led to the release of seven more drought-tolerant OFSP varieties in 2011 (Andrade et al., 2016). When RAC started, Mozambique had 15 varieties of OFSP and a number of ongoing OFSP projects. The national agriculture production study conducted in 2015, the year RAC ended, showed that 32% of sweetpotato produced in the country was orange-fleshed and people ate it two times per week (Ministério, 2015).

Improved varieties of OFSP first arrived in Tanzania in the late 1990s, mostly in the Lake Victoria zone, where sweetpotato is a primary staple food and is grown by 99% of the farming households. These varieties were distributed by CIP in all vitamin A for Africa (VITAA) partnership countries, namely Tanzania, receiving 9,259,950 cuttings; Uganda, 18,896,374 cuttings; Kenya, 12,093,920 cuttings; Mozambique, 4,621,185 cuttings; and Ethiopia, 1,691,920 cuttings. After being introduced in the Lake Victoria zone, OFSP gradually spread to the Eastern zone of Tanzania. By the time RAC started, Tanzania had released its own two OFSP varieties, namely Mataya and Kiegea. Other varieties such as Ejumula, Jewel, UKG 05 and Kabode were in trials for release. Awareness on OFSP was generally low among policymakers and investors across the country.

Nigeria is the largest root and tuber crop producing country in Africa, growing mainly yam, cocoyam, cassava and white sweetpotato. However, at the beginning of RAC in April 2011, OFSP was largely unknown in Nigeria and no variety had been released, although some on-farm and on-station trials were under way. Awareness among policymakers and investors on OFSP was very low. Therefore, RAC's efforts in year 1 (2011–2012) focused on fast-tracking the release of varieties. In year 2 farmers participated fully in the assessment of proven varieties and field days, and in year 3, two OFSP varieties, Mothers' Delight and King J, were released. In year 3 the project also focused on intensifying the selection of more decentralized vine multipliers (DVMs) and multiplication and distribution of vines in the four targeted states of Benue, Nasarawa/Federal Capital Territory in Abuja, Kwara and Kaduna. The area under OFSP continues to expand, partly because the crop has the advantage of requiring little land and few inputs. OFSP is relatively easy to grow and provides more energy per hectare and over time than rice, maize or cassava or other root crops [14], and its short maturing period of three to five months, ability to grow under marginal conditions and flexible planting and harvest times also are driving its expansion in the country.

## 2 RAC as a potential scaling up model

Armed with enough evidence base that pro-vitamin A-rich OFSP can be a cost-effective means to combat VAD in children under 5 years of age, development partners shifted attention to scaling-up of this innovative technology [15,16]. It is in this context that the debate and various action research have been conducted to determine the best model and pathways for scaling-up of the crop [16].

Scaling-up is complex [17] and there are many definitions. Scaling-up refers to any form of expansion of an intervention as a means to achieve widespread benefits and impacts for the population [18]. Frake and Messina [18] analyzed various approaches to scaling-up and posit that “scaling up requires an integrated approach, giving consideration to an integral pathway that aims at scaling up along both an institutional and geographic pathway from the onset” [18]; p. 6).

RAC project was a 3.5-year initiative (2011 and 2015), implemented in Tanzania, Mozambique, Nigeria, and to a lesser extent Ghana and Burkina Faso by the International Potato Center and Helen Keller International. RAC advocated for increased investment in orange-fleshed sweetpotato (OFSP) to combat vitamin A. RAC further built capacity at three inter-related levels: national host institutions to deliver a specialized course on OFSP through a cascading model; national experts and institutions to design and implement gender sensitive projects that drive up-take of OFSP; and strengthened capacity of African advocates and champions^1^ to engage and influence key decision makers, governments, private sector and donors to invest in OFSP projects along the value chain. RAC designed advocacy and fund-raising strategies and established a vibrant pool of multi-sectorial advocates. Thus, the scaling-up model adopted by RAC consisted of three pillars, namely a) policy change b) institutional capacity and c) proven technologies.

**Fig. 1:**
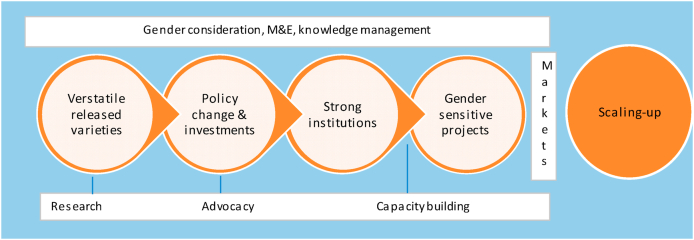
The RAC scaling-up hypothesis.1.***Versatile released varieties/technologies:*** The RAC project supported breeding priorities of national agricultural research institutions and private sector. The project facilitated the fast-tracking research in the release of pipeline varieties and the mainstreaming of OFSP in national programmes.2.***Policy change and investments:*** RAC built the capacity of diverse advocates and champions to promote and advocate for supportive national and regional policies, strategies and plans that prioritize support for OFSP/biofortification. The project focused on catalyzing the process of policy engagement for policy formulation and implementation and mobilizing new investments to fund projects on OFSP.3.**Strong institutional capacities:** The project aimed at strengthening the capacities of national and community institutions and individuals to design and implement **gender-sensitive projects** to ensure wide access and utilization of OFSP along the value chain.

The RAC experience showed that advocacy made great sense in countries where the OFSP was officially released and successfully piloted in multiple regions. Availability of multiple and versatile varieties of the crop suited for different agro-ecological zones was a huge plus. In countries where the crop was newly introduced such as Nigeria, research and efforts to accelerate the official release was given priority. With adequate varieties released, promotion and behavior change communication was given emphasis, even more than advocacy.^2^ Advocacy for policy change and resource allocation were considered necessary ingredients in scaling-up the crop. The former helped create a conducive policy environment for programming, while the latter involved engaging donor agencies (e.g. NGOs, private sector, philanthropists, federal and local governments) to invest in these programs and projects. Once resources were made available and the policy environment supportive, institutions and change agents were equipped with critical skills to implement technically strong and gender sensitive projects to spur large scale production, processing, marketing and utilization of the crop. This formed the second pillar, designated ‘institutional capacity’**.** Finally, gender considerations and dynamic, monitoring, evaluation (M&E) and knowledge management system was put in place for adaptive management and to ensure the OFSP projects benefitted both men and women. This potential scaling-up hypothesis is shown in Fig. 1.

These three pillars were augmented by crosscutting themes, namely gender, knowledge management, monitoring and evaluation. RAC recognized the role that women and youth play in agriculture and nutrition, and the project's resource mobilization strategy was not only pro-poor, but also responded to the needs of women and youth. The project therefore ensured that **gender was mainstreamed** throughout the operations. For instance, gender was mainstreamed in all the learning modules and videos were engendered, the project targeted and built the capacity of women and youth organizations (such as Shingirirai in Mozambique) to design gender sensitive projects on OFSP. The project further built and implemented a dynamic **Monitoring, Evaluation and Learning (MEL)** system that provided a framework for collecting accurate, relevant and timely information to enable the project meet information needs for learning purposes. Knowledge management practices such strict documentation of progress and lesson learned were also implemented.

This article therefore presents the achievements of the RAC project as a potential scaling-up model, highlighting the challenges and lessons learned during the implementation. The achievements, challenges and lessons were documented by an external independent ex-post evaluation consultant. This paper is therefore extracted from a larger ex-post evaluation survey report whose methods are described below.

## 3 Evaluation approach, design and methodology

This evaluation exercise used mixed methods involving a review of RAC literature, field visits and interviews with M&E officers from recent RAC-affiliated projects, field staff, national advocates, policy-makers from government departments and NGOs, representatives of training institutions, trainees, breeders, decentralized vine multipliers (DVMs) and individual farmers and processors. Ultimately, these methods were geared towards obtaining data that underpin the contribution of RAC in new policy formulation in favor of food-based approaches to combat VAD, resource allocation and improved nutrition.

In terms of attribution, for policy, only documents introduced during the RAC epoch and directly attributed to the project's effort and cited biofortification, food-based approaches and/or OFSP directly were considered. To determine attribution, the evaluator undertook a meta and content analysis of all available literature and food/agroculture/nutrition policies in the region and those drawn from the three target countries (Mozambique, Nigeria and Tanzania). Using content analysis and triangulated by key informant interviews, the evaluator analyzed the documents and teased out themes related to OFSP, biofortification and food-based approaches.

As for other results, the evaluation used the logic model as its conceptual basis to identify distinct but closely linked phases in the process of the project's service delivery: inputs, processes, outputs and outcomes [19]. The particular value of this approach is that it has an underlying systems-thinking that assists to understand the complex interactions between these elements over time. The approach also draws attention to the way in which policy is implemented and services are delivered, and how the consequences of these actions are eventually expressed. Taken together, the mixed methodology and logic model assist in identifying the inputs (resources allocated to the project), activities (how the project was implemented), outputs (types of support and services delivered), and final outcomes e.g. results and impacts. This goes beyond dealing with just the question of what works, to include consideration of why it works, under what circumstances and for whom [20]. The evaluation framework is also underpinned by social justice principles, which emphasize the importance of participatory and collaborative research. In line with hypothetical systems thinking, the evaluation defined OFSP as a system for reducing VAD in the more remote areas of Mozambique, Tanzania and Nigeria through policy and institutional development, as defined by RAC scaling up model.

The field work took place from 23 August to 14 September 2017. The actual period of data collection was four days in Mozambique and Tanzania and five days in Nigeria. In addition to interviewing 25 key persons purposively selected (10 in Tanzania, 12 in Mozambique and 13 in Nigeria), the assessor conducted focus 12 group discussions with groups of farmers and traders and visited production sites, OFSP factories and open markets in the three countries. In Tanzania, the assessor visited Sokoine University of Agriculture (SUA) and interacted with facilitators and trainees during the training of trainers (TOT) course that took place 13–24 August 2017, to understand how the training would benefit them and their communities. Besides this, a structured questionnaire focusing on capacity building and resources invested into step-down courses was emailed to the participants who had attended the TOT courses on “Everything you ever wanted to know about OFSP” in the three countries during and after RAC to find out whether they cascaded the training to the grassroots communities in their respective organizations and communities. A total of 37 ToT respondents (10 in Tanzania, 21 in Mozambique and 6 in Nigeria) submitted their dully filled questionnaires.

In documenting resources mobilized post RAC, in addition to requiring ToT beneficiaries to indicate the investments in step-down courses, respondents were asked to state whether they were aware on of any project whose funding is attributed to RAC. Using a snowball technique, the evaluator visited these projects to document resources invested in OFSP.

In spite of the limited time available for field work, the assessor triangulated various types of information collected and, in this respect found it useful to follow Lynn and Preskill [21] who seek demonstration of rigor in (1) the quality of thinking, (2) credible and legitimate claims, (3) cultural context and responsiveness, and (4) quality and value of the learning process. The assessor had access to an independent expert for support; and was familiar with the Malawian experience.

Throughout the entire data collection period, the assessor collaborated with and consistently debriefed professional colleagues familiar with the work in the target countries as part of the process to validate responses from the interviewees. Preliminary findings of the evaluation were presented on 12 October 2017 at the second annual review and planning meeting of the Building Nutritious Food Baskets (BNFB) project,^3^ where experts from CIP, the International Center for Tropical Agriculture (CIAT), International Maize and Wheat Improvement Center (CIMMYT), International Institute of Tropical Agriculture (IITA), Forum for Agricultural Research in Africa (FARA), HarvestPlus and national implementing partners provided their input. In particular, the assessor had face-to-face meetings with key experts and influencers, who were otherwise not reached during the actual data collection period. These interactions provided additional information concerning RAC and on the introduction of multiple biofortified crops for the first time by CIP and it partners, what worked, lessons learned, challenges faced, current levels of collaboration and work anticipated in the years to come.

## 4 Findings

### 4.1 Resource mobilization and investments in OFSP

At its very beginning RAC planned to generate new investments totaling US$ 18 million for OFSP activities in the three years of its life. Following the advocacy and awareness raising activities that took place before its end in August 2015, RAC's funding, at US$ 21.6 million, was 20% over its target, indicating that awareness and communications by HKI and CIP on advocacy for policy change and mobilization of resources to support programs on biofortification were effective. Mozambique's funding was the highest at US$ 13,342,550.50 followed by Tanzania's with US$ 4,033,501.50, Burkina Faso's with US$ 2,963,244, Nigeria's with US$ 1,262,479.42 and Ghana's with US$ 42,036.

As shown in Table 1, most investment came from the United Nations agencies, the private sector and local governments. The funding was used to support 52 interventions. Of the total funding, 77.8% was spent on vine multiplication and dissemination, OFSP production and processing; 7.4% on awareness and promotion in the mass media; and 14.8% on training.

**Table 1:** Breakdown of investment by donor category in Mozambique, Tanzania and Nigeria.

Type of Donor	Mozambique		Tanzania		Nigeria	
	Total (US$)	%	Total (US$)	%	Total (US$)	%
National government	–	–	234,201	5.81	1,215,332.30	96.26
Local governments	–	–	111,147.50	2.76	15,972.77	1.27
External governments/UN agencies	10,792,550	80.88	3,593,100	89.08	–	–
NGO/foundations	40,000	0.31	–	–	–	–
Private sector	2,510,000	18.81	95,053	2.35	31,174.35	2.47
**Grand total**	**13,342,550**	100	**4,033,501.50**	100	**1,262,479.42**	100

Tanzania had the most diversified sources of investments and Nigeria the least, with almost all its investment coming from the federal government. The main external donors were the African Development Bank, the Department for International Development of the United Kingdom (DFID), the International Fund for Agricultural Development (IFAD), Irish Aid, Japan International Cooperation Agency (JICA), the United States Agency for International Development (USAID) and the World Bank. These same donors have been funding the post-RAC agenda to increase uptake of OFSP and other biofortified crops. Five new OFSP projects ran by the national agencies surveyed in this evaluation received funding in the range of US$ 500,000 to US$ 12 million between 2015 and 2017. RAC trained these agencies and change agents in proposal development as well as the planning, implementation and monitoring of gender-sensitive projects.

Besides multilateral and bilateral donors, host governments have been allocating resources in annual budgets for relevant line ministries, national research institutions and provincial and district councils to continue with breeding work, farm trials, multiplication of clean vines, vine distribution and extension services. In Nigeria, it was the federal government that provided funding of US$ 819,289.34 (N134,500,000 then) for the Rainbow project, which was an offshoot of the RAC project. The Rainbow project reached well over 40,000 households. The federal government is also funding the 10-day TOT training, covering full scholarships for participants since the 2016/2017 financial year. Most recently, the federal government provided US$ 130,000 for distribution of OFSP as an emergency-response crop to address the food insecurity situation among the internally displaced people in Borno State.

During the 2017/2018 fiscal year the Tanzanian government budgeted Tanzania shillings 11 billion (approximately US$ 5 million) for children's nutrition and health activities that include OFSP, translating into 1000 shillings per child or US$ 0.44. Some provinces in Mozambique are prioritizing OFSP in the fight against VAD after the government declared it as a priority crop in 2016.

RAC expected that by the end of project period, there would an increase in vitamin A intake of at least 30% among the targeted beneficiary areas in Tanzania and Mozambique and 15% in Nigeria. Ultimately the project hoped to benefit at least 600,000 households directly and 1.2 million households indirectly within five years after project closure. This forward-looking targeting banked upon the upcoming projects making incremental coverage and impact by reaching 93,580 direct farmers annually until August 2020. By the end of 2015, RAC had reached 132,098 farmers directly with vines.

The available data reveal that recent projects are on track, having distributed clean planting materials to an additional 177,876 direct beneficiaries (of whom 20.3% were women) in the past two years, thereby reaching 95% of the two-year target.

### 4.2 The policy environment

Through its advocacy work, RAC and its advocates and champions supported the promulgation and enactment of at least 14 policy and strategic documents. These documents are listed in Table 2.

**Table 2:** Policy/strategic documents that included OFSP in the RAC primary countries.

Mozambique	Nigeria	Tanzania
1. The Comprehensive Africa Agriculture Development Programme (CAADP)/Strategic Plan for the Agricultural Sector Development (PEDSA) investment plan; 2. Socioeconomic plans for Inhambane, Maputo, Manica, Tete, Zambezia and Sofala provinces; 3. Multi-Sectoral Action Plans for Chronic Malnutrition Reduction (PAMRDC) at the provincial level; 4. The communication strategy under Multi-Sectoral Action Plans for Chronic Malnutrition Reduction (PAMRDC); 5. National food security baseline survey assessment instruments; 6. The National Child Feeding Policy; 7. The National Home Gardening Program; 8. The National School Feeding Program.	1. The Agriculture Transformation Agenda; 2. The micronutrient prevention guidelines developed by the Ministry of Health; 3. The Infant and young child feeding manual.	1. The national agriculture policy; 2. The Agricultural Sector Development Programme I; 3. The national nutrition strategy implementation plan.

The key impact of RAC’S campaigns on policy is that it positioned OFSP on top of the nutrition agenda, making it a central crop for biofortification programs. For example, before RAC the policies and strategies that had been approved by the Mozambique government for reducing malnutrition did not mention biofortification or the use of OFSP as one of the viable and cost-effective nutrition strategies. These included the Strategy and Action Plan for Food Security 2008–2015, the Strategic Plan for the Agricultural Sector Development (PEDSA) for 2010–2019, and the Action Plan for Multi-Sectoral Action Plan for the Reduction of Chronic Undernutrition in Mozambique (PAMRDC) for 2011–2015. The other documents included the Government's Five-Year Plan 2009–2014 and the Action Plan for the Reduction of Poverty (PARP) III 2011–2014. By implication, crops such as OFSP were not receiving adequate attention from donors, which affected investment in them. RAC, in partnership with SETSAN, embarked on advocacy for policy reformulation that resulted in the establishment of a communications working group and a working group on biofortification (BioSANWG) in 2013. Since then more than 15 important strategic documents have incorporated biofortification and two national programs now include OFSP as a strategic crop in Mozambique. On top of this, the role played by RAC and other partners in advocacy led to the commitment by the Government of Mozambique to invest and support production of OFSP through CAADP and the national investment plan for the agricultural sector. Another policy of significance in Mozambique is the National Agriculture Investment Plan for 2014–2018, which recognizes biofortified crops as nutritious and vital in addressing micronutrient deficiencies. Item 39 of that policy commits the Ministry of Agriculture to the following actions:

• Undertake contract programs with specialized vine and seed companies to ensure the importation and local production of the most productive certified seed varieties and vines adapted to the areas of production;
• Support the production and dissemination of clean OFSP vine material from locally based sources;
• Provide production inputs in potential sweetpotato and potato production areas.

The policy estimates the total budget for supporting the production of OFSP and potatoes to be 1.533 million meticais (US$ 25,111.18).

In Tanzania, RAC influenced the inclusion of OFSP in three (3) key policy/strategic documents. The advocacy momentum created by RAC has continued even after the project came to an end. The government has continued to include OFSP in the new policies and strategies for agriculture, food security and nutrition to combat VAD. The priority policies in Tanzania post RAC advocating for increased consumption of OFSP and other biofortified foods are:

• The National Multi-Sectoral Nutrition Action Plan (NMNAP) for 2016–2021;
• The Tanzania Food and Nutrition Centre (TFNC) five-year strategic plan for 2014–2018;
• The Food and Nutrition Security Policy of 1992, which is in its final stages of review and promulgation.

One of the planned targets for the NMNAP is to contribute to the reduction in the prevalence of vitamin A deficiency among children aged 6–59 months by 7% by 2021. Among the activities for achieving this target, the NMNAP mandates the Ministry of Agriculture, Livestock and Fisheries to promote the multiplication of seeds, seedlings and cuttings of nutrient-rich crop varieties such as OFSP, high protein maize and cassava and vitamin A rich bananas and distribute them to farmers. Among the other nutrition issues, the NMNAP emphasizes promotion and consumption of biofortified and high nutrient value food varieties at the community level to increase nutrient intake. In line with these efforts, the government has committed US$ 115 million for the implementation of the five-year NMNAP and allocated US$ 5 million in the 2017/2018 financial year budget.

Moreover, in Tanzania, issues of fortification were under the jurisdiction of the National Food Fortification Alliance (NFFA) established in 2003. Following advocacy efforts by follow-up projects, NFFA, at its ad-hoc meeting held on 28 June 2017, agreed to incorporate biofortification and revised its terms of reference. NFFA then formed the National Biofortification Task Force and instructed it to start work on 1 September 2017. The revised terms of reference state that the objective of the Task Force is “to provide to the National Food Fortification Alliance (NFFA) technical advice and recommendations on scaling up biofortification as a complimentary initiative for combating hidden hunger in Tanzania”. The National Biofortification Task Force is composed of key actors from the government and NGOs, research institutions, academia and the private sector.

In Nigeria, after RAC, follow-on projects such as the Rainbow project, Working to Improve Nutrition in Northern Nigeria (WINNN) and those implemented by Catholic Relief Services (CRS) influenced the inclusion in national plans, guidelines and manuals of biofortification, and OFSP in particular, as a food-based approach. The policies and strategic plans of significance include:

• The Nigerian Food and Nutrition Policy (2016–2020);
• The draft Nigerian Food and Nutrition Strategic Plan of Action;
• The Agricultural Sector Food Security and Nutrition Strategy (2016–2025) of the Federal Ministry of Agriculture and Rural Development's (FMARD) Innovative Agricultural Transformation Agenda.

The FMARD agenda envisions two types of value chains as paramount to its success: (1) nutrition value chains, where the main outcome is improved intake of vitamin A and other essential nutrients by school children and children under five years of age and their caregivers, and (2) the diversified product value chain, where farmers, processors and marketers are linked up productively.

### 4.3 Seed systems

RAC trained staff in government research institutions and provided them with foundation seed for farm trials, breeding and agronomic research, which resulted in the recent release of four (4) new varieties in Mozambique to make a total of 19. Three (3) other varieties are in the pipeline for official release in 2019. In Tanzania, six (6) varieties – Mataya, Kiegea, Ejumula, Kabode, UKG 05 and Kakamega – and nine (9) others are in the pipeline for official release. Nigeria has three varieties, Mothers’ Delight, Solo Gold and King J, and plans to release one (1) more variety at advanced stages.

RAC put in place an elaborate three-tier seed multiplication plan to ensure consistent and sustainable supply of OFSP seed for multiplication and production. This system is well established and working in all the three countries. The primary source is usually the research institutes that produce and supply foundation seed to the secondary multipliers, e.g. other research institutions or agricultural development programs at the regional or provincial level. The secondary multipliers in turn supply seed to tertiary level DVMs for further multiplication, all of which happens during the dry season, before distribution of the seed to farmers for planting during the rainy season. In Nigeria RAC modified this procedure and supplied foundation seed directly to DVMs, who multiplied it and supplied the vine and root producers. The agricultural development programs have limited capacity for dry season multiplication and were circumvented in this case.

To increase access to clean planting materials and decrease the risk of disease and virus contamination, RAC built the capacities of change agents on two innovative technologies for conserving seeds during the dry season, which DVMs have started using. In areas with high virus pressure, trained DVMs are using net tunnels to maintain a stock of disease-free planting material sourced from research stations before bulking the vines in wetlands close to the beginning of the rainy season. In areas with dry periods lasting over four months storing small roots in sand and sprouting them in protected beds is being used successfully. This procedure is known as the Triple S (storage in sand and sprouting) method. One such sprouted root generates 40 cuttings for planting at the beginning of the rains. In Mozambique these technologies are being experimented with and used by DVMs in Niassa, Manica and Sofala, which are major sweetpotato growing areas. Other follow-up interventions such as the Kinga Marando project run by the Lake Zone Agricultural Research and Development Institute and CIP in Tanzania and the SASHA project in Nigeria also have continued to use the technologies and are promoting them to farmers.

### 4.4 Capacity development for key actors

#### 4.4.1 Capacity development for national institutions

Between January and June 2012, RAC identified national institutions to collaborate with in the delivery of the 10-day TOT course on “Everything you ever wanted to know about sweetpotato” on an annual basis during and after the project. Various agriculture research and management training institutions and universities were evaluated for suitability. The Eduardo Mondlane University in Mozambique, the SUA Department of Agribusiness and Agricultural Economics based in Morogoro in Tanzania, and the Agricultural and Rural Management Training Institute (ARMTI) in Ilorin, Kwara State, Nigeria were selected.

RAC worked through a mentorship process with these institutions to ensure that they had the right capacity to deliver the course. Before each 10-day course, a pre-training workshop was conducted where national facilitators were paired with experienced RAC, CIP and HKI scientists for the different modules. The team developed session plans and rehearsed the course delivery process. Also covered were the TOT delivery methodology, facilitation skills, adult learning methodologies, and gender issues in OFSP, among other topics. Clarification was made on how to use the course manual before the actual training, and the learning-by-doing activities were planned. The facilitators ensured that all training materials required during the course were available. The pre-training workshop took five days in the first round, but the duration was gradually reduced during the second and third rounds, based on evolving needs.

In Tanzania five-day workshops were held in November 2012 and July 2013, and a two-day workshop in March 2014. Five days were needed in the second round owing to the addition of a relatively large number of new national facilitators from various departments of the university, who constituted six of the 20 participants. In Nigeria five-day workshops were held in November 2012 and September 2013, and a three-day workshop was held in June 2014. During the first workshop, relatively few CIP scientists were available because it coincided with the 10-day TOT course in Tanzania, which was in its second week. Some CIP facilitators had to remain in Tanzania for that course.

In Mozambique the pre-training workshop took five days in the first round during July to August 2012 and two days in the second round in July and August 2013. No formal pre-training occurred during the third round but two new facilitators were coached individually.

The capacity of the national facilitators was strengthened further through the stepwise planning and delivery approach adopted for the TOT courses. Taken together, the workshops and the backstopping support enhanced the knowledge and capacity of the national institutions to organize, host and facilitate step-down training for change agents from the national implementing partners.

**Fig. 2:**
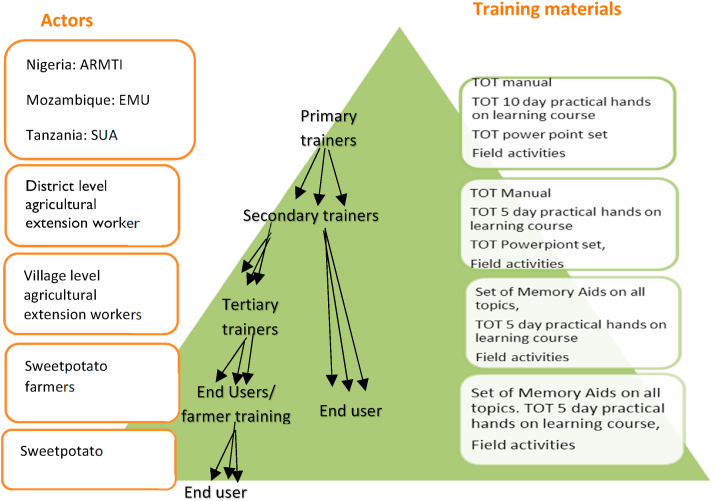
The cascading training approach adopted by the RAC project.

The three institutions trained district level government and NGO agricultural extension workers who then cascaded the training to similar cadres at the village level for the final dissemination to farmers and traders (see Fig. 2). RAC offered full scholarships for 20 participants per TOT course and made provision for another 10 privately sponsored trainees.

Before each 10-day course a pre-training workshop was conducted, where national facilitators were paired with experienced RAC, CIP and HKI scientists for the different modules. At the beginning of the training each participant received the TOT manual. The manual helped to guide the training and served as the key reference document. At the end of the training each participant received a branded CD containing all the training materials used by the facilitators during the course, including PowerPoint presentations, photos, recipes and other reference documents for use during subsequent step-down training.

In addition to the three primary host institutions, RAC built the capacity of 51 national agencies in the three countries. In most cases trainees went ahead to build the capacity of their own institutions to design and implement gender-sensitive OFSP projects. Examples of such institutions were the Sugarcane Research Institute (SRI) in Kibaha, the Research Community and Organizational Development Associates (RECODA), the Agricultural Research Institute (ARI) in Hombolo and ARI–Kizimbani in Tanzania; Shingirirai in Mozambique; and Partnership for Child Development in Nigeria.

#### 4.4.2 Continuation of the TOT course by national institutions

##### 4.4.2.1 Nigeria

This evaluation exercise involved consulting institutions identified by RAC to establish whether the 10-day TOT courses were still taking place in the three countries. The study found that ARMTI had institutionalized and integrated the TOT course fully in its curriculum. ARMTI is a parastatal organization under the Federal Ministry of Agriculture and Rural Development of the Federal Government of Nigeria. Situated in Ilorin, ARMTI is a center of excellence in agricultural and rural development management training and human resource development. By the time of this evaluation, ARMTI had 13 trainers – of whom 4 were women – and 7 were trained by RAC in 2012.

After RAC was phased out, ARMTI's first attempt to run the course in March 2015 on a cost-recovery basis did not succeed. There were no funded applicants despite extensive advertisement for the course and subsidized tuition fees. The second attempt in October 2015 yielded some success with six participants, one of whom was female. In the 2016/2017 financial year, the federal government provided 16.5 million naira (then equivalent to US$ 54,635.76) for the training, covering transport fare, tuition, food, accommodation and stipend for the participants. ARMTI trained a total of 59 trainers in two cohorts, one from 21 November to 2 December 2016 and the other one in March 2017. The participants were from the Agriculture Development Program, universities, research institutes, NGOs and the private sector. They were drawn from the six states identified as the main producers of sweetpotato, i.e. Kwara, Osun, Ebonyi, Kaduna, Benue and Nasarawa. Some 22% of the participants were female.

##### 4.4.2.2 Tanzania

By the time of this evaluation, SUA had conducted one 10-day TOT course with sponsorship from CIP's VISTA project, for district agriculture, extension and nutrition officers. The course was split into two 5-day modules, which were offered 6–10 July and 18–21 August 2015. Thirty-four participants attended module 1, of whom were 13 female, and 32 of them were present during module 2, of whom 12 were female.

SUA conducted another 10-day TOT during 13–24 August 2017 for 27 participants (37% females), mainly district level, local government and NGO extension and health staff (Table 3). Some of the participants were supported by World Vision and the BNFB project. The aim was to build institutional and community capability in biofortified crops.

**Table 3:** Breakdown of participants in RAC TOTs from 2012 to 2017.

Year	Nigeria			Tanzania			Both Countries
	Sex			Sex			
	M	F	Total	M	F	Total	Overall
2012^a^	12	8	20	18	12	30	50
2013^a^	17	11	28	13	10	23	51
2014^a^	21	10	31	16	7	23	54
2015^b^	5	1	6	20	12	32	38
2016^c^	25	7	32	3	3	6	38
2017^c^	16	5	21	17	10	27	48
**Total (n)**	**96**	**42**	**138**	**87**	**54**	**141**	**279**
**%**	69.6	30.4	100	61.7	38.3	100	100

a Conducted with RAC funding.

b Conducted on cost-recovery basis in Nigeria and funded by CIP's VISTA project in Tanzania.

c Conducted under 2016 ARMTI's capital project and cost-recovery basis, and for Tanzania funded by CIP's VISTA project for 2016. In the 2017 SUA TOT course participants partially sponsored by World Vision and the BNFB project.

##### 4.4.2.3 Mozambique

Eduardo Mondlane University had not facilitated any training over the period September 2015 to August 2017. The respondents attributed this to funding problems associated with the national economic recession facing the country. Calls for self-sponsored participants had attracted little or no interest.

##### 4.4.2.4 Stepping down of training

After training the national institutions, RAC expected cascading of the knowledge and skills to the grassroots to allow wider uptake and utilization of OFSP. This evaluation found that majority of the people who had received TOT training as secondary trainers were continuing to train others in their organizations, districts and communities. Figures from the 38 secondary facilitators indicate that a total of 71,602 people had been trained through step-down courses (Table 4). Participants stated that:

“The course content and the style of the training are very good. I liked the group work exercise and the interaction of the facilitators and trainees. It made the class very active and most importantly it enabled us to share experiences to learn new ways of doing things (TOT participant from Agricultural Research Institute in Tanzania).”

**Table 4:** Stepping down of TOTs from 2015 to 2017 by trainers interviewed in the evaluation.

District	Organization	RAC ToTs interviewed			Trainees trained by ToTs through step-down			
					Trainees	Sex		
		M	F	Total		M	F	Total
Nigeria	Government departments and research institutions	4	0	4	Agriculturists, extension officers, farmers and processors	245	250	495
	NGOs	0	3	3	Farmers	959	2237	3196
	**Total**	**4**	**3**	**7**		**1204**	**2487**	**3691**
Tanzania	Government departments and research institutions	2	5	7	Agriculturists, extension workers, nutritionists, school pupils, farmers and processors	14,033	30,508	44,541
	NGOs	2	1	3	Farmers	7613	12,018	19,631
	**Total**	**4**	**6**	**10**		**21,646**	**42,526**	**64,172**
Mozambique	Government departments and research institutions	**11**	**1**	**12**	Extension workers, nutritionists, students, farmers and processors	770	608	1378
	NGOs	3	0	3	Nutritionists and farmers	607	1620	2227
	Private sector	4	0	4	Farmers	73	56	129
	Academia	2	0	2	**Students**	4	1	5
	**Total**	**20**	**1**	**21**		**1454**	**2285**	**3739**
**Overall**		**28**	**10**	**38**		**24,304**	**47,298**	**71,602**

Generally, financial support was considered the main drawback:

“If I plan to train extension officers and farmers from my district I need money for lunch, per diem, stationery, venue and transport during and after the training to follow up or monitor progress. The local government that sent me to attend the TOT has no budget to support such activities, and in most cases we do these trainings informally with farmers. Trainers of end-users also require training materials such as processing equipment and start-up vines for distribution to the new farmers trained, and additional support such as backstopping services and information, education and communication materials” (TOT participant from Tanzania).

Similar sentiments were echoed by trainers from Nigeria.

In Mozambique most of the step-down training events were funded by various projects run by different NGOs, Christian and farmers’ associations and government departments. These included Samaritans Purse, CIP, Asociación Madre Coraje, HKI, Economic Agents, provinces, Christian Association Fund, Institute for Agriculture Research of Mozambique (IIAM), Olima Wo Suka project, Union of Cooperatives and Agricultural Associations of Lichinga, Agribusiness Consortium of Chimoio, Kenmare Moma Development Association and Niche Project, which is funded by the Dutch Cooperation.

### 4.5 Gender mainstreaming in RAC activities

Throughout its lifespan, RAC ensured gender was mainstreamed in its operations. For example, gender was a major selection criterion for identifying course participants. The intention was to have female and male participants in almost equal proportions, for both women and men to have access to knowledge and skills about OFSP that would benefit their children. Tanzania had the most gender-balanced attendance in the 10-day TOT course and 6-day training course on project planning, implementation, monitoring and evaluation with females constituting 51.4% of the 3000 trainees. It was followed by Nigeria, with 39% of the 415 trainees as female, and then Mozambique with females constituting 37.6% of the 1019 trainees. The participants observed that the 10-day course was not attractive to females because it kept them away for too long from their household responsibilities.

Other strategies that RAC used to uphold gender equality in its operations included ensuring gender mainstreaming in the learning toolkits and other learning materials as well as in the log frames and monitoring indicators in the project proposals. Aside from this, during identification of DVMs, RAC made a deliberate effort to include women so that they too had access to quality vines. Routine monitoring data from the project shows that the household member obtaining OFSP from RAC's primary and secondary sites was 76.4% female in Mozambique, 49.6% female in Tanzania and 12% female in Nigeria, where OFSP had been newly introduced. Also, where necessary, RAC supported female commercial vine multipliers along with their male counterparts with foundation seed, irrigation equipment and linkage with buyers.

In Africa women are the main producers of sweetpotato and also make the decisions on food choices in the homes. For OFSP, women dominate in all activities and men become involved when the scale of operation is large. The use of OFSP to combat VAD, therefore, makes sense because those most at risk are children in poor households where women are the dominant caregivers and decision-makers in food preparation. This central role of women also adds importance to the well-known link between gender and nutrition and provides a unique opportunity for education and training of women in the utilization of OFSP from production through to cooking and serving in the home.

To ensure a gender balance, the TOT course covers all aspects of production and use of OFSP for both male and female farmers. Efforts have also been made by follow-up projects to involve school children in school gardens, where they gain hands-on experience in production of OFSP and ways of preparing it at home, and in school feeding programs.

## 5 Constraints for OFSP

In spite of the successes achieved, RAC and its follow-up projects are not without challenges, as new constraints have emerged that affect the universal adoption and utilization of OFSP and even other biofortified products. These challenges are discussed below.

### 5.1 Preference for the high dry matter content of traditional varieties despite their lack of beta-carotene

In all the three countries studied, farmers and consumers continue to prefer the traditional sweetpotato varieties with high dry matter and low water content. The high dry matter content in white-fleshed sweetpotatoes is valued or vital for filling the stomach of the consumer. OFSP is considered softer in texture after cooking even for varieties that breeders categorize as high in dry matter. Sadly, the darker the orange color and richer in beta-carotene OFSP is, the softer the texture. Plant breeding is going on to create OFSP varieties with high dry matter content and the preferred sensory characteristics to increase its adoption. Meanwhile, advocacy messages should focus on mindset and behavior change so that OFSP is not regarded as similar to or a replacement for white sweetpotato, so that it is adopted for its nutritional benefits, which are absent in the white varieties.

### 5.2 Seasonality, perishability and storage problems

Sweetpotatoes are generally available from May through October in the three countries. Different storage methods are being promoted for the roots: (1) pit storage containing layers of dry grass and leaves, (2) sawdust heap, (3) rack storage, (4) in-ground storage where at root maturity the vines are cut off, the soil hilled up to cover exposed roots and the cracks closed up, which can store the roots for two to three months, and (5) dry sand storage under a ventilated room at room temperature, which can store the roots for four months and longer.

Fresh roots can be stored in dry sand for five months, but this practice is not common in real life. Farmers prefer pit storage as it resembles the traditional structure (see a sample in the photo below) used for storing white-fleshed sweetpotatoes, which they are used to. The main problem is that OFSP roots can be stored for only up to three months using this method before they wrinkle and rot. Maintaining the roots in the field after maturity results in heavy weevil infestation within as brief a period as one month.

Breeders and scientists are aware of the storage problem but coming up with methods that can store fresh roots for long periods is complicated by the need to maintain the stability of beta-carotene during storage. A study by Jenkins et al. [16] found that beta-carotene begins to decline after 12 weeks of indoor storage and 22 weeks of in-ground storage. Bechoff et al. (2011) found high losses of carotenoids associated with storage of dried OFSP chips, leading to a recommendation that OFSP chips be stored for no longer than two to four months, depending on the variety.

Invigorating the processing and commercialization of OFSP at a large scale can in part help address the need for long-term storage of fresh roots. In large scale undertakings, for instance, instead of storing fresh roots, OFSP could be processed into puree for incorporation into flour products to reduce the costs of importing wheat flour, or dried in different forms for storage in Purdue PICS bags for periods of three years and longer.

**Figure:**
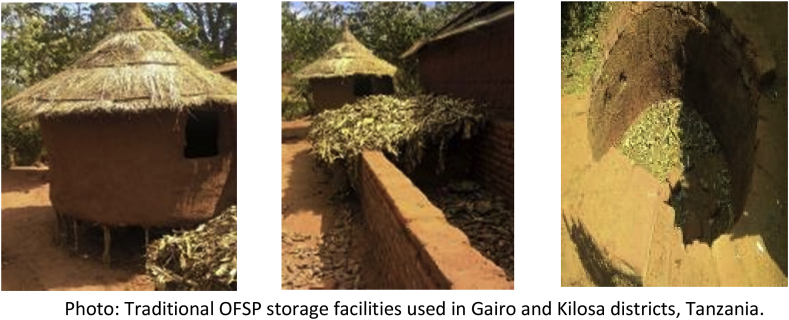

### 5.3 Prolonged and recurring dry spells

Sweetpotato's primary multiplication starts during the dry season to generate vine cuttings for secondary multiplication during the rainy season. RAC promoted the use of net tunnels and Triple S methods to conserve OFSP vines during the dry season. Despite the adoption of these innovative technologies, by the time of this evaluation majority of the farmers were still relying on traditional methods of multiplying vines in wet lands, which are tedious and dependent on a very good supply of water. Therefore, vine multiplication was limited to small parcels of land of 0.25 acres to 1.5 acres. It was reported in all the three countries that because of this reason there were many times when the demand for vines was much higher than the supply, which negated the work of the campaigns and promotion activities undertaken. The erratic rains and prolonged dry spells in the 2013 to 2016 agricultural seasons complicated the situation.

## 6 Lessons learned and the way forward

Below are some lessons learned in scaling-up OFSP in SSA and the way forward:

### 6.1 Value chains and markets for OFSP are poorly developed and fragmented

In all the three priority countries of RAC, value chain actors for OFSP and white-fleshed sweetpotato are the same. They include input suppliers, farmers, transporters, traders, processors, retailers and consumers. In its design, RAC focused mostly on advocacy for policy change and resource mobilization and capacity building, not on linkages to markets or agroprocessing for value addition. It assumed that different actors would be willing to participate in the value chain voluntarily and that market links would be made through other initiatives. However, production has remained largely small scale on land sizes of 1 acre to 1.5 acres and for home consumption. Farmers give priority to cereals and other crops they perceive to have high market demand and higher profits. The low production of OFSP limits its supply to non-farming households in both rural and urban areas that are net buyers of food. It also prevents large-scale traders and marketers from making meaningful investments in it. In short, OFSP has not really penetrated the main urban markets for wider consumer utilization. It is available mainly in markets in its areas of production and only during the production season. Farmers, who serve as both producers and traders, lack market-related information on prices, value chains, competitors and credit. A large majority of them have no market contacts and face difficulties finding potential buyers.

Other issues include infrastructural inadequacies and behavior and attitude factors, with the general perception of sweetpotato as a women's and disaster-response crop owing to its drought tolerance. Traders in urban markets have better market information and stronger linkages with processors but bulking is a challenge because, like farmers, they operate as individuals. This, along with the production constraints at the farm level, limits the demand for OFSP processed products. The lack of financial support has resulted in low involvement of processors. Notwithstanding these bottlenecks and given the current interest in nutrition and the favorable policy environment brought about by RAC, the potential exists for an improved and well-functioning value chain environment and markets that will enable key players to derive greater benefits from their activities.

### 6.2 Successful promotion of biofortified crops requires cross-sectional collaboration at the country level

After successfully implementing RAC, in BNFB CIP embraced a paradigm shift from one to multiple crops and value chain environment, both of which require strong leadership and clear definition of roles to enable multiple agencies with different expertise, knowledge and networks to work together in a synergistic manner. The need for effective coordination is particularly evident because most of the crops in the BNFB project are relatively new and have different ecological and protocol requirements. This evaluation found more than adequate guidance from RAC and the BNFB project that future interventions can learn from. For example, the operation approach of the coordination framework has improved relationships between the partner agencies, interagency communication and awareness on other biofortified crops. In addition, the activities of the projects, the commitment of the partner agencies to achieving results, and the partners’ willingness to work outside of rigid operating hours have contributed to the successful coordination of the project at the country level.

The BNFB project has shown that every commodity is *sui generis*, the process for each crop varies with commodity sector and country, and for biofortified crops the critical thing is not biofortification per se but market development. The future will bring into play several new factors: competing biofortified staple crops, new potential value chains, and the need to bridge technical, institutional and market gaps. CIP has accumulated experience, established many links and developed several manuals and TOT materials. The knowledge and resources produced have adaptability possibilities for other commodities and environments; for example, they can be used in institutional analysis and human capacity development aspects, and they are highly pertinent in maintaining CIP as the logical backbone organization in future biofortification programs.

CIP's pioneering work on biofortification of sweetpotato began with collaboration with CIAT and the International Food Policy Research Institute (IFPRI) and in two stages: IFPRI and HarvestPlus proof of concept and reaching end-users. The work in the three countries that are the subjects in this study was at a commodity-specific level, but CIP has built capacity that has proved useful to other commodities, including white-fleshed sweetpotato.

### 6.3 Successful scaling-up require strong linkages in health, nutrition and agriculture

Going forward, research institutions such as CIP should continue to embrace the necessity of improved linkages between agriculture and health and nutrition without sacrificing technical work. Low productivity in agriculture, particularly among smallholder farmers, is the main reason for the low penetration of biofortified products and their low intake, which contributes to undernutrition. Farmers should be supported to have access to the inputs they need to ensure high and sustainable production of quality biofortified food commodities. They need improved storage facilities to reduce the unacceptably high levels of postharvest losses and secure access to markets. Appropriate and affordable financial products ought to be developed, especially for smallholders and more so women farmers, who depend on their own production for most of their food and for whom increased productivity is a sure route out of poverty. This support is needed even more in the face of climate change to maximize the benefits of agricultural development and to make communities more resilient.

## 7 Conclusion

As a potential scaling-up model, RAC succeeded in moving from resting on its success in the technical aspects of the project to the development of necessary knowledge and understanding of agents of change and building the capacity of individuals and institutions to promote biofortified OFSP. In doing so it developed linkages with other centers and NGO partners with similar interests. Moreover, although OFSP already existed in Mozambique and Tanzania before RAC, a large majority of farmers lacked it and were still growing traditional white varieties without beta-carotene or other crops. RAC scaled up the breeding and distribution of clean OFSP planting materials and consumption and value addition activities for this crop in these countries.

In terms of scaling up, according to Bouis and Islam [22]; a country's OFSP program may be in any of the three stages. Stage 1 countries are characterized by farm level consumption, a critical mass of poor farmers adopting a biofortified crop and feeding it to their families. Stage 2 involves market development to provide farmers with an outlet for their marketable surplus, thus reaching non-farming or rural households that are the net buyers of food. This stage is driven by the need to reach out to medium-scale producers and to develop local markets and demand for products made from biofortified crops, though still largely in the rural areas. Stage 3 countries are those that have embraced modern value chains. The private sector in these countries are the main drivers of the diffusion process and they develop modern value chains to produce value-added tradable products. Based on this study, Mozambique, with the longest involvement and experience in OFSP, is currently in stage two. Nigeria is a major sweetpotato producer, ranking second after China, but OFSP is new there, having been introduced by RAC in 2012. Tanzania is striving to get into stage two. The progress of a country into the next stage is contingent upon the continuation of investments by both its host and external governments, capacity building for the local structures, an enabling environment for different key actors, active involvement of the private sector, and effectiveness of awareness campaigns for the majority of farmers and consumers to adopt the crop.

## Footnotes

1 These were regional or country-level actors and/or entities with a passionate commitment to the fight against malnutrition, ill health, and food insecurity, and with interest and strong potential as advocates for the dissemination and use of OFSP as a means to reduce VAD and improve food security. The OFSP champions/advocates hailed from a variety of disciplines and sectors, including NGO and GO sectors, academia and civil society, as well as from the agricultural, health, or human development sectors.

2 Whereas advocacy refers to public support to influence decisions for policy change and resource allocation within institutions and national systems, promotion refers to a set of activities that communicate, create awareness and enhance demand for the product to the end user. However, where possible, with adequate resources, advocacy and promotion can be conducted simultaneously with emphasis varying according to how much the biofortified crop is already established.

3 The BNFB project was designed to test a scaling-up model for biofortified crops. The project was built on the achievements, success, and scaling up approaches of the RAC project and drew on complementary CGIAR expertise to demonstrate a scaling up model for biofortified crops. The BNFB project therefore broadened focus from OFSP to a multi-crop (“food basket”) approach.
